# Interference of Dihydrocoumarin with Hormone Transduction and Phenylpropanoid Biosynthesis Inhibits Barnyardgrass (*Echinochloa crus-galli*) Root Growth

**DOI:** 10.3390/plants11192505

**Published:** 2022-09-26

**Authors:** Haona Yang, Shangfeng Zhou, Lamei Wu, Lifeng Wang

**Affiliations:** 1 Hunan Weed Science Key Laboratory, Hunan Agricultural Biotechnology Research Institute, Hunan Academy of Agricultural Sciences, Changsha 410125, China; 2 Hunan Rice Research Institute, Hunan Academy of Agricultural Sciences, Changsha 410125, China

**Keywords:** barnyardgrass, dihydrocoumarin, botanical herbicide, oxidative stress, transcriptomic, plant hormone, phenylpropanoid biosynthesis

## Abstract

Botanical compounds with herbicidal activity exhibit safety, low toxicity, and low chances of herbicide resistance development in plants. They have widespread applications in green agricultural production and the development of organic agriculture. In the present study, dihydrocoumarin showed potential as a botanical herbicide, and its phenotypic characteristics and mechanism of action were studied in barnyardgrass [*Echinochloa crus-galli* (L.) P.Beauv.] seedlings. The results indicated that dihydrocoumarin inhibited the growth of barnyardgrass without causing significant inhibition of rice seedling growth at concentrations ranging between 0.5 and 1.0 g/L. Additionally, dihydrocoumarin treatment could cause oxidative stress in barnyardgrass, disrupt the cell membrane, and reduce the root cell activity, resulting in root cell death. Transcriptomic analyses revealed that dihydrocoumarin could inhibit barnyardgrass normal growth by affecting the signal transduction of plant hormones. The results showed significant differential expression of plant hormone signal transduction genes in barnyardgrass. Additionally, dihydrocoumarin interfered with the expression of numerous phenylpropanoid biosynthesis genes in barnyardgrass that affect the production of various vital metabolites. We speculate that the barnyardgrass growth was suppressed by the interaction among hormones and phenylpropanoid biosynthesis genes, indicating that dihydrocoumarin can be applied as a bioherbicide to control barnyardgrass growth in rice transplanting fields.

## 1. Introduction

The development of environmentally friendly methods to manage plant pests can contribute to sustainable agriculture [1]. Plant allelopathy and allelochemicals are vital for the study of botanical herbicides and show a potential for sustainable and organic agriculture [2,3,4]. Allelochemicals extracted or secreted from more than 111 plants can be used to control 78 types of weeds in various crop and non-cultivated fields [5], and these chemicals include compounds such as phenols, terpenoids, organic acids, alkaloids, and quinones [5,6]. Allelochemicals are released by plant leaching, litter decomposition, root exudations, and direct plant volatilization [7]. Allelochemicals generated by litter decomposition can affect seed germination, seedling growth, and population development [8]. Allelopathy reduced recipient plant performance by 25%, whereas plant residues were more negative than the allelopathy plant leachates [9]. A field experiment was conducted to analyze the decomposition of cereal rye (*Secale cereale* L.), which was used as a cover crop for weed control; phenolic acids were identified as the main allelochemical released into the soil [10]. Our previous study revealed that the extracts of decomposed *Myosoton aquaticum* (L.) Moench could be used as a cover crop to suppress weeds; the study identified dihydrocoumarin as the main allelochemical that exerted strong inhibitory effects on barnyardgrass [*Echinochloa crus-galli* (L.) P.Beauv.] seedlings [11].

Dihydrocoumarin is a benzopyran derivative that possesses unique biological and pharmacological activities and is found in several natural products [12]. It can be synthesized by the hydrogenation of coumarin. Coumarin causes phytotoxicity to plant root growth and shows a potential hormone-like activity, particularly the auxin-like activity, and thus interferes with the root growth of *A. thaliana* [13,14]. However, Bru Saleh and Abu EI-Soud [15] reported that coumarin produced a gibberellin-like effect. Additionally, coumarin influenced the recipient growth by causing an imbalance in the endogenous hormone content. Coumarin delayed seed germination by reducing the GA4 content in *Brassica parachinensis* L.H. Bailey seeds [16] and by inhibiting abscisic acid (ABA) catabolism in rice (*Oryza sativa* L.) seeds [17]. However, coumarin is banned because of its safety risk, and dihydrocoumarin has been used as a food additive for more than 20 years [18]. Its maximum acceptable concentration in an aerosol air freshener is 1.5%; it neither exhibits mutagenicity and genotoxic potential nor poses any risk to the aquatic environment [19]. Therefore, dihydrocoumarin shows both safety and low toxicity, which are the characteristics of an ideal biological herbicide.

Barnyardgrass is regarded as one of the most harmful weeds in paddy fields that causes an average annual rice yield loss of approximately 40–60% [20]. The yield of rice could be reduced by more than 70% under the long-term influence of barnyardgrass, whereas it could be increased by 750 kg/ha when the occurrence of barnyardgrass was reduced by more than 90% [21]. Despite the rapid development of barnyardgrass resistance, biological herbicides can effectively relieve the development of resistant barnyardgrass. Dihydrocoumarin showed stronger inhibitory effects on the root growth of barnyardgrass seedlings than coumarin [11]; however, the allelopathy effect of dihydrocoumarin has been understudied. Root growth is affected by several factors [22]. Plant hormones such as auxin, cytokinin (CTK), ethylene, and ABA are involved directly in the regulation of plant root development [23,24]. These hormones regulate plant growth and allow plants to adapt to the environment at a small concentration, thus, studying their signal transduction pathway is vital to understanding their functions and the regulation mechanism in plants under various environmental stresses [25]. The phenylpropanoid metabolism is one of the most important metabolism in plants, and continuously increasing information has revealed that the phenylpropanoid metabolism is regulated at multiple levels, involving phytohormones and biotic and abiotic stresses at every growing period [26]. Plant root development is also affected by the crosstalk between signals such as reactive oxygen species (ROS) [27], nitric oxide, nutrition, and secondary metabolites generated from the phenylpropanoid pathway, such as flavonoids [28,29].

The present study evaluated the physiological response of barnyardgrass seedlings to dihydrocoumarin and explored the action mechanism of dihydrocoumarin through transcriptomic analyses and enzyme assays, providing new ideas and methods for the development of the green control of barnyardgrass in paddy fields.

## 2. Results

### 2.1. Barnyardgrass and Rice Seedling Bioassay

**Figure 1:**
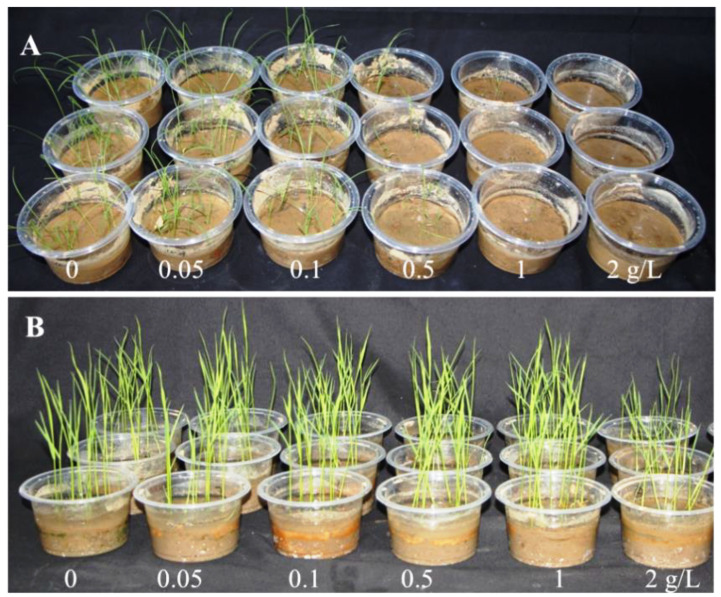
The effects of dihydrocoumarin on the growth of barnyardgrass (**A**) and rice seedling (**B**).

The effects of dihydrocoumarin on the growth of barnyardgrass and rice seedlings were determined using the pot experiment (Table 1 and Table 2). The barnyardgrass seedling growth was completely inhibited by dihydrocoumarin treatment at 2 g/L concentration. The shoot growth and fresh weight were 4.37 cm and 12.33 mg, respectively, which were significantly inhibited at 0.5 g/L. The shoot length and fresh weight of the rice were significantly decreased at 2 g/L, and no significant difference was observed between the control and other treatments (Figure 1).

### 2.2. Dihydrocoumarin Effects on Barnyardgrass Growth

**Figure 2:**
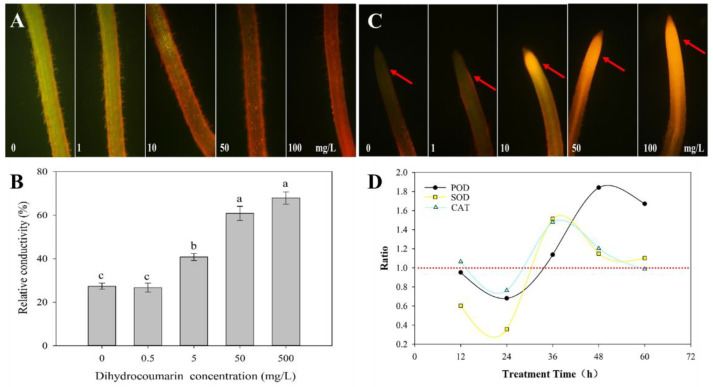
The effects of dihydrocoumarin on the growth of barnyardgrass. (**A**) The effects of dihydrocoumarin on the cell viability of barnyardgrass root. (**B**) The effects of dihydrocoumarin on the relative conductivity of barnyardgrass root. (**C**) The effects of dihydrocoumarin on ROS production in barnyardgrass roots. The arrows point to the luminous tissues of the root tip. (**D**) Contrast of controls and treatments on the antioxidant enzyme activities of barnyardgrass when treated with dihydrocoumarin at 50 mg/L. The ratio is the average enzyme activity of the treatment group divided by the simultaneous average enzyme activity of the control group.

The cell viability of the barnyardgrass root was evaluated by double staining with propidium iodide (PI) and fluorescein diacetate (FDA) after the treatment with different concentrations of dihydrocoumarin. Green and red fluorescence reflect viable and dead cells, respectively. The root cells (Figure 2A) reflected green fluorescence similar to those of the control when treated with 1 mg/L of dihydrocoumarin for 24 h. The root viability was partially lost at a concentration of 10 mg/L, and only a small part of the root cells reflected green fluorescence. The root cells reflected red fluorescence when treated with 50 and 100 mg/L of dihydrocoumarin, indicating that most of the root cells lost viability under these treatments.

Cell permeability possibly increased after the damage caused to the cell membrane, and the extravasation of various water-soluble substances resulted in an increase in the electrolyte permeability. There was no significant difference in the relative conductivity between the control group (27.32%) and the group treated with 0.5 mg/L of dihydrocoumarin (26.68%) (Figure 2B). When the barnyardgrass root was treated with dihydrocoumarin at 5, 50, and 500 mg/L concentrations, the relative conductivity gradually increased to 40.81%, 60.86%, and 67.89%, respectively, which were significantly higher than that of the control group. The electrolyte leakage increased from the barnyardgrass cell under higher dihydrocoumarin concentrations. No significant difference in the relative conductivity was observed between the roots treated with 50 and 500 mg/L of dihydrocoumarin.

The brighter orange fluorescence reflected by the barnyardgrass root tip indicated a high amount of ROS generated by the roots (Figure 2C). The barnyardgrass root tip reflected little fluorescence, indicating less ROS production in the control treatment. After treatment with 1 mg/L of dihydrocoumarin, only the fringe of the root tip reflected a weak orange florescence (the fringe tissue of the root is pointed by the arrow in the second picture of Figure 2C). The root tip that was treated with 10 mg/L of dihydrocoumarin for 24 h emitted a bright orange fluorescence, and it showed a brighter orange fluorescence after treatment with 50 and 100 mg/L of dihydrocoumarin, which meant a mass of ROS was gathered in the root.

### 2.3. Effect of Dihydrocoumarin on the Activity of Antioxidant Enzymes in Barnyardgrass Seedlings

The change trends of the SOD, POD, and CAT activities were different under the treatment with 50 mg/L of dihydrocoumarin; however, all antioxidant enzymes showed a higher activity in the treated plants than in the controls at most time points (Table S2). The ratio of the enzyme activity of the treatment groups and control groups was calculated to compare the enzyme activity between the treatment group and the control group at the same time. A ratio > 1 indicates higher enzyme activity in the treatment group than in the control group at the same detection time. Similarly, a ratio < 1 indicates a greater inhibition of the enzyme activity in the treatment group than in the control group. As shown in Figure 2D, the POD, SOD, and CAT activities were first inhibited and then increased. The activities of the three antioxidant enzymes were most significantly inhibited 24 h after dihydrocoumarin treatment. The activities of POD, SOD, and CAT were 0.68, 0.36, and 0.76 of the control, respectively. After 24 h, the enzyme activities increased; the activities of the SOD and CAT were increased to 1.51 and 1.48 times the control treatment 36 h after the dihydrocoumarin treatment, respectively, whereas the POD activity was increased to 1.84 times at 48 h.

### 2.4. Transcriptomic Analysis

After removing the low-quality reads, the RNA-seq produced 47,440,394; 48,891,217; and 49,659,251 clean reads from the control treatment libraries at 0 h (ZL00), 24 h (ZL024), and 48 h (ZL048), respectively. Additionally, it produced 47,782,675 and 46,394,379 clean reads from the dihydrocoumarin treatment libraries at 24 h (ZL24) and 48 h (ZL48), respectively (Table S3). The Q30 and GC percentages were 92.68–94.65% and 53.61–55.08%, respectively. The overall sequencing error rate was <0.03% (Table S3). The principal component analysis showed that the data within the group were highly concentrated; whereas the intergroup data were separate (Figure S1). Additionally, the Pearson correlation analysis between the samples indicated a correlation coefficient of >0.882 in the same treatment, which also confirmed the quality of biological repeatability (Figure S2). The comparison groups ZL24:ZL024 and ZL48:ZL048 exhibited 4890 (2408 upregulated and 2482 downregulated) and 20,287 (9873 upregulated and 1041 downregulated) differentially expressed genes (DEGs), respectively (Table S4).

**Figure 3:**
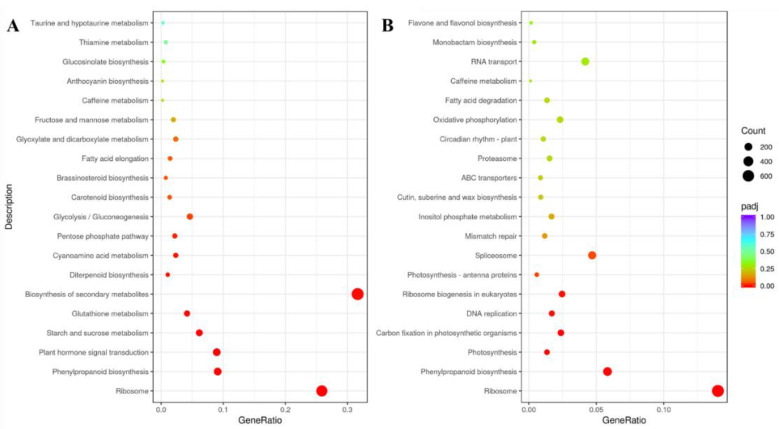
KEGG enrichment scatter diagram. (**A**) KEGG enrichment scatter diagram 24 h after dihydrocoumarin treatment. (**B**) KEGG enrichment scatter diagram 48 h after dihydrocoumarin treatment.

The Gene Ontology (GO) analyses indicated that the DEGs were mainly involved in the biological process (BP) and molecular function (MF) 24 h after the dihydrocoumarin treatment, especially the oxidative stress response and endogenous stimulation and hormone response, and the number of upregulated genes was higher than that of the downregulated genes (Figure S3). However, a reverse trend was observed with the biological process 48 h after the dihydrocoumarin treatment, and the number of downregulated genes was higher than that of the upregulated genes (Figure S4). The Kyoto Encyclopedia of Genes and Genomes (KEGG) pathway enrichment analysis revealed ribosome, phenylpropanoid biosynthesis, plant hormone signal transduction, the starch and sucrose metabolism, and the glutathione metabolism to be the significantly enriched pathways in the ZL24:ZL024 comparison (Figure 3A). In the ZL48:ZL048 comparison, the significantly enriched pathways of the DEGs were the ribosome, phenylpropanoid biosynthesis, photosynthesis, carbon fixation in photosynthetic organisms, and the other four pathways (Figure 3B). The KEGG enrichment trend was similar to that of the GO enrichment. Several DEGs were related to the BP and MF, whose expression was upregulated 24 h after dihydrocoumarin treatment and downregulated after 48 h, leading to the biological function disorders of cells in barnyardgrass seedlings. Additionally, the expression of the DEGs of ribosome and DNA replication pathways was downregulated after 24 h and upregulated after 48 h, probably because dihydrocoumarin mainly acted on the BP of the barnyardgrass seedlings and inhibited various normal transcription functions. This feedback promoted MF gene expression.

In the experiment of transcriptomic analysis, 50 mg/L of dihydrocoumarin significantly inhibited the growth of the barnyardgrass root, and no significant difference was observed between the control and treatment groups 48 h after germination (Figure S5). The observed phenotype of barnyardgrass root echoed the physiological results and indicated that the action mechanism of the dihydrocoumarin was associated with root development. According to the comprehensive analysis of KEGG and the GO enrichment results, combined with the physiological results and the DEGs, we further analyzed the plant hormone signal transduction pathway and phenylpropanoid biosynthesis pathway of barnyardgrass seedlings after the dihydrocoumarin treatment.

#### 2.4.1. Plant Hormone Signal Transduction Pathway

**Figure 4:**
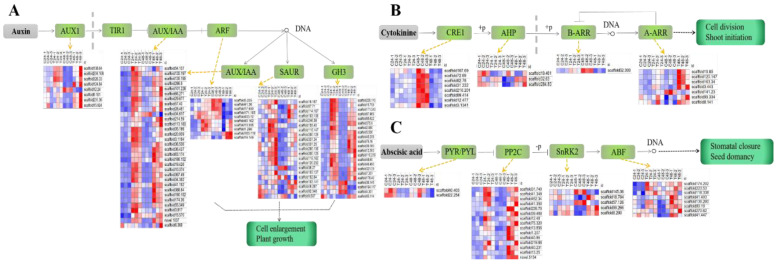
Heat map of the differentially expressed genes of the plant hormone signal transduction pathway. The different expressed genes are defined as |log2FoldChange| > 1 and Padj < 0.05. (**A**) is auxin signal transduction pathway, (**B**) is cytokinin signal transduction pathway, and (**C**) is abscisic acid signal transduction pathway. C24-1, C24-2, and C24-3: the control group at 24 h (three repetitions); C48-1, C48-2, and C48-3: the control group at 48 h (three repetitions); T24-1, T24-2, and T24-3: the treatment group (50 mg/L dihydrocoumarin) at 24 h (three repetitions); and T48-1, T48-2, and T48-3: the treatment group (50 mg/L dihydrocoumarin) at 48 h (three repetitions).

The KEGG Pathviews of ZL24:ZL024 and ZL48:ZL048 showed that the signal transductions of the plant auxin, CTK, and ABA were significantly enriched in the plant hormone signal transduction pathway (Figure S6). The DEGs (|Log_2_FoldChange| > 1 and Padj < 0.05 at both 24 h and 48 h) of these hormone signal transduction pathways were selected, and the fragments per kilobase per million (FPKMs) of these DEGs were normalized (Z-score) for the heat map analysis. In the present study, the expression of the DEGs, namely *AUX1*, *AUX/IAA*, *SAUR*, and *GH3*, was mostly upregulated in the auxin transduction pathway, whereas the expression of most of the *ARF* DEGs was upregulated first at 24 h and then downregulated at 48 h (Figure 4A). The |Log_2_FoldChange| of the *AUX/IAA* genes, *scaffold367.49*, *scaffold190.152*, and *scaffold388.64*, was >4 at two detection time points, and the expression of these genes was significantly upregulated after the dihydrocoumarin treatment. Figure 4B illustrates the DEGs of the CTK transduction pathway. The expression of the cytokinin receptor CRE1 protein and B-ARR DEGs was significantly downregulated, and the downregulated AHP and A-ARR protein genes were more abundant than the corresponding upregulated genes. The *CRE1* genes of *scaffold12.477* and *scaffold72.69* were downregulated more than six times 48 h after the dihydrocoumarin treatment. ABA plays a crucial role in plant development and participates in the regulation of a plant’s response to stress. In the ABA transduction pathway (Figure 4C), the downregulated PYR/PYL protein genes were more abundant than the corresponding upregulated genes, whereas the upregulated *SnRK2*, *PP2C*, and *ABF* genes were more abundant than the corresponding downregulated genes.

#### 2.4.2. Phenylpropanoid Biosynthesis Pathway

**Figure 5:**
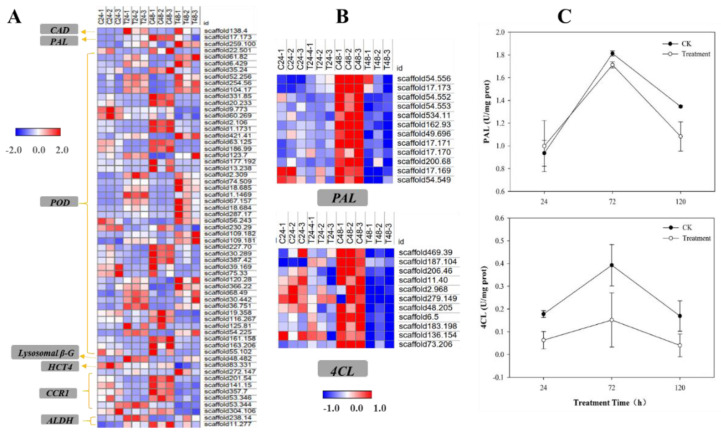
Dihydrocoumarin interferes with phenylpropanoid biosynthesis. (**A**) Differentially expressed gene (DEG) heat map of the phenylpropanoid biosynthesis pathway. The DEGs are defined as |log2FoldChange| > 1 and Padj < 0.05; *CAD:* cinnamyl alcohol dehydrogenase, *PAL*: phenylalanine ammonia lyase, *POD*: peroxidase, *Lysosomal β-G*: lysosome β-glucosidase, *HCT4*: hydroxycinnamoyl transferase 4, *CCR1*: cinnamoyl-CoA reductase 1, and *ALDH*: acetaldehyde dehydrogenase. (**B**) DEG heat map of *PAL* and *4CL*. *4CL*: 4-coumarate-CoA ligase. The DEGs are defined as |log2FoldChange| > 1 and Padj < 0.05 at 24 h or 48 h; (**C**) dihydrocoumarin effects on PAL and 4CL activities of barnyardgrass at 50 mg/L. The data are expressed as the mean ± SE. C24-1, C24-2, C24-3: the control group at 24 h (three repetitions); C48-1, C48-2, C48-3: the control group at 48 h (three repetitions); T24-1, T24-2, T24-3: the treatment group (50 mg/L dihydrocoumarin) at 24 h (three repetitions); and T48-1, T48-2, T48-3: the treatment group (50 mg/L dihydrocoumarin) at 48 h (three repetitions).

The DEGs were significantly enriched in the phenylpropanoid biosynthesis pathway from the KEGG pathway analysis of ZL24:ZL024 and ZL48:ZL048 (Figure S7). Upregulated genes were more abundant 24 h after the dihydrocoumarin treatment, whereas downregulated genes were more abundant 48 h after the dihydrocoumarin treatment. At both detection time points, 61 DEGs were significantly regulated, and the FPKM values of these DEGs were normalized (Z-score) and analyzed to generate a heat map (Figure 5A). The results revealed 1 cinnamyl alcohol dehydrogenase (*CAD*) gene (*scaffold138.4*), 1 phenylalanine ammonia lyase (*PAL*) gene (*scaffold17.173*), 48 peroxidase genes (*POD*), 1 lysosomal beta glucosidase (*lysosomal β-G*) gene (*scaffold48.482*), 1 hydroxycinnamoyl transferase 4 (*HCT4*) gene (*scaffold83.331*), 7 cinnamoyl-CoA reductase (*CCR1*) genes (*scaffold272.147*, *scaffold201.54*, *scaffold141.15*, *scaffold357.7*, *scaffold53.346*, *scaffold304.206*, *scaffold53.344*), and 2 aldehyde dehydrogenase (*ALDH*) genes (*scaffold238.14*, *scaffold11.277*). The phenylpropanoid biosynthesis pathway is a complex one that involves multiple proteins, and its regulation is controlled by several key enzymes [26]. PAL, C4H, and 4-coumarin CoA ligase (4CL) are three key enzymes in the phenylpropanoid biosynthesis pathway, and most of the genes related to these three key enzymes could not meet the required conditions (|Log_2_FoldChange| > 1 and Padj < 0.05 at both 24 h and 48 h) for DEGs. Thus, these genes are not presented in Figure 5A. Figure 5B illustrates the DEGs (Padj < 0.05 at 24 h or 48 h) of the key enzymes PAL and 4CL. Most of the DEGs of the PAL and 4CL enzymes showed a significantly decreased expression at 48 h. However, no significant difference was observed in the expression of the *C4H* genes between the control and dihydrocoumarin treatment. The expression of four *PAL* genes, namely *scaffold54.552*, *scaffold54.553*, *scaffold534.11*, and *scaffold49.696*, was substantially inhibited, and the expression in the control treatment exceeded five times that in the dihydrocoumarin treatment at 48 h. Additionally, the expression of the three *4CL* genes, *scaffold206.46, scaffold73.206*, and *scaffold11.40*, increased by four times at 48 h.

### 2.5. qRT-PCR Validation for Transcriptomic Analysis

To verify the accuracy of the RNA-seq data, 12 DEGs of the plant hormone signal transduction pathway and phenylpropanoid biosynthesis pathway were selected, including 3 *PAL* genes (*scaffold54.552*, *scaffold54.553*, and *scaffold534.11*) and 3 *4CL* genes (*scaffold206.46*, *scaffold73.206*, and *scaffold11.40*). The results are illustrated in Figure S8. The qRT-PCR results were similar to the RNA-seq results, indicating the same general expression trends. Both upregulated and downregulated genes in the qRT-PCR assay were consistent with those in the RNA-seq results.

### 2.6. PAL and 4CL Activities

The results of RNA-seq and qRT-PCR indicated that the expression of the *PAL* and *4CL* genes was downregulated after treatment with 50 mg/L of dihydrocoumarin. The activities of PAL and 4CL were determined using the test kits in barnyardgrass seedlings after the dihydrocoumarin treatment to validate the protein expression of the downregulated genes. The activity of the enzymes PAL and 4CL first increased and then decreased under both control and dihydrocoumarin treatments, indicating that the addition of 50 mL of water and dihydrocoumarin probably stimulated the activities of PAL and 4CL to resist stress. The increase in enzyme activities was higher after the dihydrocoumarin treatment than after the control treatment, which suggests that the enzyme activities were probably suppressed by dihydrocoumarin at the same detection time points. These enzymes were most active at 72 h after the dihydrocoumarin treatment, with the activities being 1.71 and 0.15 U/mgprot, respectively (Figure 5C). The PAL activity of the treatment group was 6.62% higher than that of the control group 24 h after the dihydrocoumarin treatment. Additionally, 4CL was more significantly inhibited at any detection time point after the dihydrocoumarin treatment compared with that under the control treatment, although it increased at 48 h and decreased to nearly 0 after 96 h. The results confirmed that dihydrocoumarin affected the production of several vital metabolites by inhibiting the key enzymes involved in the phenylpropanoid biosynthesis pathway.

## 3. Discussion

The present study evaluated the bioherbicidal potential of dihydrocoumarin for controlling barnyardgrass growth in rice fields. In this study, treatments with 0.5–1.0 g/L of dihydrocoumarin were found to be safe for rice seedlings (3- to 4-leaf stage) and useful for controlling barnyardgrass seeds, which indicated the efficacy of dihydrocoumarin in controlling barnyardgrass growth in rice transplanting fields.

ROS are the widespread signaling molecules that regulate a series of plant physiological processes and play a vital role in various signaling pathways [30]. External environmental stress promotes ROS accumulation in plants, resulting in ROS imbalance. Excessive ROS generation leads to the destruction of cell structure and DNA division, and induces programmed cell death in plants [31,32]. We found the root tip was obviously reflecting bright orange florescence than the control under the treatment of 10 mg/L of dihydrocoumarin, that meant this treatment increased much more ROS production. The treatment with 5 mg/L of dihydrocoumarin significantly increased the relative conductivity in the barnyardgrass root, indicating that the cell membrane was disrupted and the water-soluble substances were extravasated form the cell. The treatment with 10 mg/L of dihydrocoumarin eventually decreased root cell viability, caused root cell death, and inhibited root growth. SOD, POD, and CAT are the efficient ROS scavengers that avoid the toxicity to plants under biotic and abiotic stresses [33]. The treatment with allelochemicals causes adverse external stress to the recipient plants, which usually leads to ROS accumulation in plant cells and the inhibition of the activities of various protective enzymes [34]. The results proved that the barnyardgrass seedlings exhibited oxidative stress and ROS accumulation after the dihydrocoumarin treatment, which stimulated the barnyardgrass antioxidant enzyme activities to resist stress.

Several allelochemicals cause ROS accumulation, which results in the disordered transduction of plant hormones, and eventually inhibits the growth of plant root. Thymol induced an increase in the ABA and H_2_O_2_ contents of *A. thaliana* [35]. As a signaling molecule, ROS is accumulated by external environmental stress and stimulates some key pathways to adapt to stress in the plant. The quercetin treatment significantly induced the rice root growth inhibition and disturbed the plant hormone signal transduction [36]. Benzoic acid and *p*-cumaric acid treatments did not significantly upregulate the expression of *Aux/IAA* of wild peanut as that of cultivated peanut, which rendered the wild peanut root more susceptible and led to its more severe growth inhibition [37]. Transcriptomic analysis showed that the rice root elongation was inhibited by ferulic acid which regulates the dynamic balance between the hormones ethylene and jasmonic acid [38]. Additionally, the transcriptomic analyses showed that when there were DEGs in the auxin, the CTK and ABA signal transduction pathways were significantly upregulated in barnyardgrass after the dihydrocoumarin treatment, indicating that the significant upregulation or downregulation of several key proteins might regulate the primary root growth of barnyardgrass. Many overexpressed genes such as *Aux/IAA*, *AUX1*, *SAUR*, and *GH3* affected the auxin transduction and inhibited the auxin regulation in response to the adverse stress of the dihydrocoumarin treatment, which inhibited the normal growth of the barnyardgrass root. External environmental factors regulate the *Aux/IAA* function [39], which is also involved in the stress and defense responses [40]. External stress also mediates the differential expression of *GH3* and *SAURs* [41]. Song et al. [42] observed that numerous *Aux/IAA* genes were related to the establishment of the rice root, and an overexpressed *OsIAA1* impeded the improvement in the rice root growth. The CTK response regulator of A-ARR inhibited the B-ARR expression as a negative regulator and blocked subsequent CTK transcription. The overexpression of *ARR5* and *ARR7* has been reported to improve the *A. thaliana* tolerance to low temperature and drought [43,44]. Additionally, CTK was reported to be associated with Al stress and root growth inhibition [45]. The dihydrocoumarin treatment resulted in the upregulation of DEGs involved in the CTK transduction pathway; thus, we speculate that the barnyardgrass acquired stress resistance by increasing the expression of A-ARR genes. The ABA receptor PYL can inhibit the expression of the SnRK2 protein activated by osmotic stress, and the ABA signal transduction mediated by PYL is necessary for a plant response to abiotic stress [46]. ABA signal transduction was reduced by the inhibition of the PYR/PYL and SnRK2 proteins expressions after the dihydrocoumarin treatment of barnyardgrass seedlings. Different hormones interact with each other and form a complex signal network system, thereby jointly participating in plant stress regulation. However, the interactions among different hormones mediated by dihydrocoumarin must be studied further.

In addition to hormone transduction, the biosynthesis of phenylpropanoid was also affected by the dihydrocoumarin treatment, as evident from the results of transcriptomics analyses. The expression of the *PAL* and *4CL* genes was obviously downregulated in the phenylpropanoid biosynthesis pathway, and the dihydrocoumarin treatment influenced the activities of the enzymes, suggesting that dihydrocoumarin showed inhibitory effects on the key enzymes involved in phenylpropanoid biosynthesis, namely PAL and 4CL. The phenylpropanoid biosynthesis pathway is one of the main secondary metabolic pathways in plants that generate phenylpropanoid metabolites such as flavonoids and terpenoids, which are crucial to plant defense and adaptation to the environment [47]. PAL and CCR1 expressions were significantly upregulated in the resistant *Phytophthora capsici*, suggesting that the biosynthesis of phenylpropanoid played an important role in *Phytophthora capsici* resistance [48]. It has been reported that plant growth is affected by the interaction between phenylpropanoid metabolites and different hormones [26]. Heat stress caused an increase in the PAL activity in pea (*Pisum sativum*), thus improving the synthesis of salicylic acid and stimulating plant immunity to heat stress [49]. In the present study, we speculate that barnyardgrass growth was suppressed by the interaction among hormones and phenylpropanoid biosynthesis genes.

## 4. Materials and Methods

### 4.1. Materials and Plant Treatment

The barnyardgrass seeds were collected from a sensitive population of Dingchen (DC) in the Hunan Province [50]; a single plant was used for propagation, and all plants had a relatively consistent genetic background. The rice (Huanghuazhan) seeds were purchased from a store. All barnyardgrass and rice seeds were sterilized with 75% alcohol and 3% sodium hypochlorite solution. The seedlings were cultivated in a light incubator at a 10 h photoperiod at 28 °C under dark conditions and then at a 14 h photoperiod at 30 °C under a light intensity of 100 μmol m^−2^ s^−1^.

The barnyardgrass seeds were cultured with 8 mL of deionized water in a Petri dish. After 72–96 h, the barnyardgrass roots grew to approximately 1.0 cm. The barnyardgrass seedlings with similar shoot and root lengths were selected and treated with 100, 50, 10, 1, and 0 mg/L of dihydrocoumarin. After 24 h, the seedlings were washed with deionized water to remove the dihydrocoumarin on the root surface. Then, the seedlings were used for the analyses of cell viability and ROS production. The seedlings were cultivated in a similar manner with 500, 50, 0.5, and 0 mg/L of dihydrocoumarin and were used for the analysis of cell membrane permeability. Based on the previous research, the barnyardgrass seedlings were cultivated using the agar method for the determination of enzyme activities and transcriptomic analyses [11]. The seeds were sown on the surface of 100 mL of 0.25% agar in a 250 mL plastic cup, and 40 mL of 50 mg/L of dihydrocoumarin or water was added after 48 h. Root growth in the treatment groups was seriously inhibited, while shoot growth was not significantly inhibited compared with that of the controls (Figure S5), which was useful to explore the action mechanism of dihydrocoumarin affecting the growth of the barnyardgrass root. Seedlings were collected 12, 24, 36, 48, and 60 h after treatment for antioxidant enzyme activity determination. For PAL and 4CL activity determination, seedlings were collected 24, 72, and 120 h after treatment, whereas for transcriptomics analysis, seedlings were collected 24 and 48 h after treatment. Three replicates were prepared for each treatment group, and all seedlings were frozen quickly with liquid nitrogen and stored in a refrigerator at −80 °C.

Dihydrocoumarin with a purity of 99%, PI and FDA with a purity of 97%, and dihydroethidium (DHE) with a purity of 95% were purchased from Shanghai Macklin Biochemical Co., Ltd. (Shanghai, China). Calcium chloride (CaCl_2_), acetone, ethanol, Tween 80, and sodium hypochlorite were purchased from Sinopharm Chemical Reagent Co., Ltd. (Shanghai, China). A total superoxide dismutase (SOD) test kit (WST-1 method), peroxidase (POD) test kit, catalase (CAT) test kit (ammonium molybdate method), and total protein (TP) test kit (Coomassie brilliant blue method) were purchased from Nanjing Jiancheng Bioengineering Institute (Nanjing, China). The PAL and 4CL activity assay kits were purchased from Beijing Solarbio Science and Technology Co., Ltd. (Beijing, China).

### 4.2. Bioassay for Barnyardgrass and Rice Seedlings

A total of 15 sterilized barnyardgrass and rice seeds were planted in disposable plastic cups (68 × 80 mm; 200 mL). After 2 days, 40 mL of dihydrocoumarin was added to the barnyardgrass cups. The amount of water lost was replenished every 2 days for 2 weeks, and the shoot length and fresh weight of barnyardgrass seedlings were measured after 2 weeks. Similar treatments were performed in rice cups after nearly 2 weeks (3- to 4-leaf stage). The shoot length and fresh weight of the rice seedlings were measured 5 days after the treatment. The experiment was repeated three times and each experiment was used as three biological replicates. The concentrations of dihydrocoumarin were 2000, 1000, 500, 100, 50, and 0 mg/L.

### 4.3. Cell Viability Analyses

The cell viability of the barnyardgrass roots was determined by double staining the root tissues with PI and FDA using a technique described by Pan et al. [51] with some modification. Five root tissues were stained for 10 min with a mixed solution dye of FDA (12.5 μg mL^−1^) and PI (5 μg mL^−1^), and then observed under a florescence microscope after removing the dye on the surface. Three biological replicates were collected from every treatment group. The fluorescence was measured at the excitation and emission wavelengths of 488 and 510 nm, respectively. The dead cells emit red fluorescence, whereas the viable cells emit green fluorescence.

### 4.4. ROS Production Analyses

The ROS level was determined with DHE in the barnyardgrass roots [52]. Five barnyardgrass roots were cut for staining with a mixed solution dye of 100 mM DHE, CaCl_2_, and 0.01% acetone (pH 4.7) for 10 min in the dark. Then, the barnyardgrass roots were soaked in 100 mM of CaCl_2_ for 20 min to remove the residual dyes. The roots were observed under a florescence microscope after washing to remove the dye on the surface. Three biological replicates were collected from every treatment group. The fluorescence was measured at an excitation wavelength of 488 nm and an emission wavelength of 510 nm. The roots emitting brighter fluorescence were considered to have a high ROS content.

### 4.5. Cell Membrane Permeability Analyses

The membrane permeability of barnyardgrass root cells was determined by measuring electrolyte leakage using the method described by Zabalza et al. [53]. Ten barnyardgrass roots were cut and soaked in a test tube with 10 mL of ultra-pure grade water at 25 °C. After soaking the roots for 2 h, the initial conductivity (E0) was determined using a conductivity meter (DDS-11A, Shanghai INESA Scientific Instrument Co., Ltd., Shanghai, China). Then, the test tube was heated in boiling water for 15 min, which inactivated the barnyardgrass roots. The water lost by evaporation was replenished to 10 mL after the test tube was cooled to 25 °C, and the final conductivity (Et) was then measured. Three biological replicates were collected from every treatment group. The electrolyte leakage was calculated as the ratio of E0 and Et (E0/Et). A higher ratio indicates a high degree of damage to the barnyardgrass roots treated with dihydrocoumarin.

### 4.6. Determination of the Activities of the Antioxidant Enzymes, PAL and 4CL

The barnyardgrass seedlings were ground and homogenized with liquid nitrogen. Then, the enzyme activity and TP content were determined using assay kits following the manufacturer’s instructions. The experiments were repeated three times and each experiment was used as three biological replicates.

### 4.7. Transcriptome Sequencing

The total RNA of the barnyardgrass seedlings was extracted using the DP441 Total RNA Purification Kit (TIANGEN, Beijing, China), whereas the RNA integrity was assessed using the RNA Nano 6000 Assay Kit of the Bioanalyzer 2100 system (Agilent Technologies, Santa Clara, CA, USA). The mRNA was purified from the total RNA by using poly-T oligo-attached magnetic beads. The RNA sequencing (RNA-seq) library preparation, clustering, and sequencing of the samples were performed using the method described by Wang et al. [54] The prepared libraries were sequenced on an Illumina HiSeq platform, according to the manufacturer’s instructions (Illumina, San Diego, CA, USA). The barnyardgrass reference genome and gene model annotation files were downloaded from the website directly (http://ibi.zju.edu.cn/RiceWeedomes/Echinochloa/)(accessed on 7 November 2021) (*E. crus-galli* [v. 2.0]). The reference genome index was built, and paired-end clean reads were aligned to the reference genome by using Hisat2 (v. 2.0.5). The mapped reads of each sample were assembled using a StringTie (v. 1.3.3b) by using a reference-based approach, whereas featureCounts (v. 1.5.0-p3) was used to count the reads mapped to each gene.

### 4.8. Differential Expression Analysis

Differential expression analysis was performed using the DESeq2 R package (v. 1.20.0). The DEGs with |log_2_FoldChange| ≥ 1 and adjusted-P (Padj) ≤ 0.05 were selected. GO and KEGG enrichment analyses for the DEGs were implemented using the clusterProfiler (v. 3.4.4) package.

### 4.9. Real-Time Quantitative Polymerase Chain Reaction Verification

The extracted total RNA was reverse-transcribed using the reverse transcription kit (A2791, Promega, Madison, WI, USA), and the entire 20-μL reaction system was adopted in this project. A real-time quantitative polymerase chain reaction (qRT-PCR) verification was performed on 12 DEGs according to the transcriptome results. The primer information is listed in Table S1. Referring to the barnyardgrass reference gene screened by our research group, the *UBQ* gene was selected as the reference gene [55].

### 4.10. Statistical Analysis

Three biological replicates were collected from every treatment group, and the data are presented as the mean ± standard error for the three replicates. The statistical analysis was conducted using SPSS (v. 26.0) software (IBM, Armonk, NY, USA). One-way analysis of variance was used for comparison between the groups, and the significance was determined using the Duncan’s multiple range test. A *p* value of <0.05 was considered to denote the statistical significance.

## 5. Conclusions

In summary, the barnyardgrass growth was significantly inhibited and the rice seedling growth was non-significantly inhibited under the treatment of dihydrocoumarin at 0.5 to 1.0 g/L. This study confirmed that dihydrocoumarin caused the accumulation of ROS in the barnyardgrass root, disrupted the root cell membrane, and reduced root cell activity, eventually leading to the inhibition of barnyardgrass root growth. The results of transcriptomic analyses indicated that dihydrocoumarin regulated the hormone signal transduction and phenylpropanoid biosynthesis pathways, which eventually affected the barnyardgrass growth. However, it is needed to further study the interaction among these two pathways and ROS in the barnyardgrass seedlings under the treatment of dihydrocoumarin in the future. The results obtained could provide useful information for the sustainable weed management method.

## Figures and Tables

**Table 1:** The effects of dihydrocoumarin on rice seedling growth.

Concentration (g/L)	Fresh Weight (g)	Shoot Length (cm)
0	0.79 ± 0.08 a	15.41 ± 0.85 a
0.05	0.81 ± 0.04 a	15.81 ± 1.13 a
0.1	0.78 ± 0.06 a	14.16 ± 0.82 a
0.5	0.78 ± 0.07 a	15.04 ± 0.51 a
1	0.70 ± 0.09 ab	13.93 ± 0.44 a
2	0.55 ± 0.04 b	10.76 ± 0.87 b

Data are expressed as the mean ± SE, and different letters indicate significant differences between different treatments (*p* < 0.05).

**Table 2:** The effects of dihydrocoumarin on barnyardgrass seedling growth.

Concentration (g/L)	Fresh Weight (mg)	Shoot Length (cm)
0	38.00 ± 3.21 a	8.67 ± 0.24 a
0.05	38.33 ± 3.28 a	8.43 ± 0.20 a
0.1	30.00 ± 1.00 b	8.15 ± 0.61 a
0.5	12.33 ± 0.88 c	4.37 ± 0.62 b
1	5.33 ± 2.40 d	1.53 ± 0.69 c
2	0.00 ± 0.00 d	0.00 ± 0.00 d

Data are expressed as the mean ± SE, and different letters indicate significant differences between different treatments (*p* < 0.05).

## Supplementary Materials

The following supporting information can be downloaded at: https://www.mdpi.com/article/10.3390/plants11192505/s1, Figure S1: principal component analysis of different samples; Figure S2: Pearson correlation heat map between different samples; Figure S3: GO statistical histogram of differentially expressed genes between ZL24 and ZL024 under dihydrocoumarin treatment; Figure S4: GO statistical histogram of differentially expressed genes between ZL48 and ZL048 under dihydrocoumarin treatment; Figure S5: the barnyardgrass was treated with 50 mg/L dihydrocoumarin after 24 h (A) and 48 h (B); Figure S6: plant hormone signal transduction KEGG pathways of barnyardgrass treated with dihydrocoumarin after 24 h (A) and 48 h (B); Figure S7: phenylpropanoid biosynthesis KEGG pathways of barnyardgrass treated with dihydrocoumarin after 24 h (A) and 48 h (B); Figure S8: expression of representative genes validated by qRT-PCR in barnyardgrass; Table S1: primer sequences of genes used for quantitative RT-PCR verification; Table S2: effects of dihydrocoumarin on the activities of antioxidant enzyme of barnyardgrass at 50 mg/L; Table S3: summary of sequencing results; Table S4: differential gene expression analysis among different treatments.
