# Supplementary material for: Interference of Dihydrocoumarin with Hormone Transduction and Phenylpropanoid Biosynthesis Inhibits Barnyardgrass (Echinochloa crus-galli) Root Growth
Source: Plants (Basel). 2022 Sep 26;11(19):2505. doi: 10.3390/plants11192505 (PMC9572682; doi:10.3390/plants11192505)
Supplement: Supplementary file 1 [file plants-11-02505-s001.zip › plants-1853278-supplementary.pdf]

## Supplementary Tables and Figures

**Table S1.** Primer sequences of genes used for quantitative RT-PCR verification.

| Genes | Forward primer | Reverse primer |
|---|---|---|
| *scaffold12.477* | TCAAGGTGTTGTGTAGAAAGCAATC | TTGAGCCATAAGTTCATAAACGAGC |
| *scaffold10.374* | TGAACCAAGCAACAAGAGGAAGAG | ATCCCTTCCATCTTCACCTTCAC |
| *scaffold67.469* | AATCAATGATGGTTGAAAAGCTGGA | TGTCATACAAGTCCAACAAAGCATC |
| *scaffold304.106* | TATAAGGGAGGGAGCAAATGTGAAA | TGTCGTTGTAGTCCACGAAGATTAG |
| *scaffold18.684* | GCTGGACAACCAGTACTACAAGAA | GCCTTGAATTTATCTTCCCACCAC |
| *scaffold287.17* | CAGCAACGTCAACTTCGATATGC | CTAGATGAGCTGGGGAAGGTTGAAT |
| *scaffold54.552* | AGCTCATGTCCTCGACGTTC | CTCAGGGTCTTCTTCGCCAC |
| *scaffold54.553* | CGACAACCCCTTGATCGATGTC | CCGTTGTTGTAGTAGTCGTTGAC |
| *scaffold534.11* | GCTCGTGAACGACTTCTACAACAA | CTCTGGACGTGGTTGGTGAT |
| *scaffold206.46* | GGAAACAAGTCGAGCCAGAAATC | TCTTGTAGTAGATCACCTCCTTTGC |
| *scaffold73.206* | CACAACTGATGACTTCTCGTCATTC | TTAGTTCCCTGAACTCCAGTAATCG |
| *scaffold11.40* | AGGTGATCTACTACAAGAAGGTACG | TGCTGTTGTTGAAGTTGTTTCCTTA |
| *UBQ* | CAGACCAGCAGCGCTTGA | TTACTGGCCACCACGGAGA |

**Table S2.** Effects of dihydrocoumarin on the activities of antioxidant enzyme of barnyardgrass at 50 mg/L.

| Time | POD | | SOD | | CAT | |
|---|---|---|---|---|---|---|
| (h) | CK | Treatment | CK | Treatment | CK | Treatment |
| | U $mgprot^{-1}$ | | | | | |
| 12 | 62.09±6.53 d | 59.08±8.98 d | 7.52±0.57 a | 4.54±0.31 b | 31.38±1.71 de | 33.4±0.90 cd |
| 24 | 100.83±1.21 d | 68.68±5.06 d | 5.45±0.32 b | 1.95±0.27 c | 34.38±2.26 cd | 26.24±1.25 d |
| 36 | 168.38±2.04 b | 191.57±17.70 b | 3.46±0.49 bc | 5.24±0.53 b | 31.21±0.95 de | 46.18±0.38 a |
| 48 | 125.22±14.92 c | 230.49±21.77 a | 4.14±0.14 b | 4.76±0.32 b | 34.84±0.10 cd | 41.93±0.68 b |
| 60 | 106.82±8.41 c | 178.50±18.47 b | 4.96±0.40 b | 5.47±0.63 b | 37.77±2.40 bc | 37.27±0.19 bc |

Values are expressed as the mean ± SE, and different letters within each control and treatment indicate significant differences between different treatments at different times ($P < 0.05$).

**Table S3.** Summary of sequencing results.

| Sample | Clean reads | Error Rate (%) | Q20 percentage (%) | Q30 percentage (%) | GC content |
|---|---|---|---|---|---|
| ZL00 | 47,440,394 | 0.03 | 97.37 | 92.68 | 54.22 |
| ZL024 | 48,891,217 | 0.03 | 97.84 | 93.96 | 54.91 |
| ZL048 | 49,659,251 | 0.03 | 97.81 | 93.87 | 55.08 |
| ZL24 | 47,782,675 | 0.02 | 98.12 | 94.65 | 54.1 |
| ZL48 | 46,394,379 | 0.03 | 97.9 | 94.02 | 53.61 |

Q20: percentage of the basic in all basics which phred is > 20; Q30: percentage of the basic in all basics which phred is > 30. ZL00: at 0 h, ZL024: the control group at 24 h, ZL048: the control group at 48 h, ZL24: the treatment group (50 mg/L dihydrocoumarin), ZL48: the treatment group (50 mg/L dihydrocoumarin) at 48 h.

**Table S4.** Differential gene expression analysis among different treatments.

| Groups | Upregulated genes | Downregulated genes | Total DEGs |
|---|---|---|---|
| ZL24vsZL024 | 2,408 | 2,482 | 4,890 |
| ZL48vsZL048 | 9,873 | 10,414 | 20,287 |

ZL024: the control group at 24 h, ZL048: the control group at 48 h, ZL24: the treatment group (50 mg/L dihydrocoumarin), ZL48: the treatment group (50 mg/L dihydrocoumarin) at 48 h.

## Supplementary Figure Legends

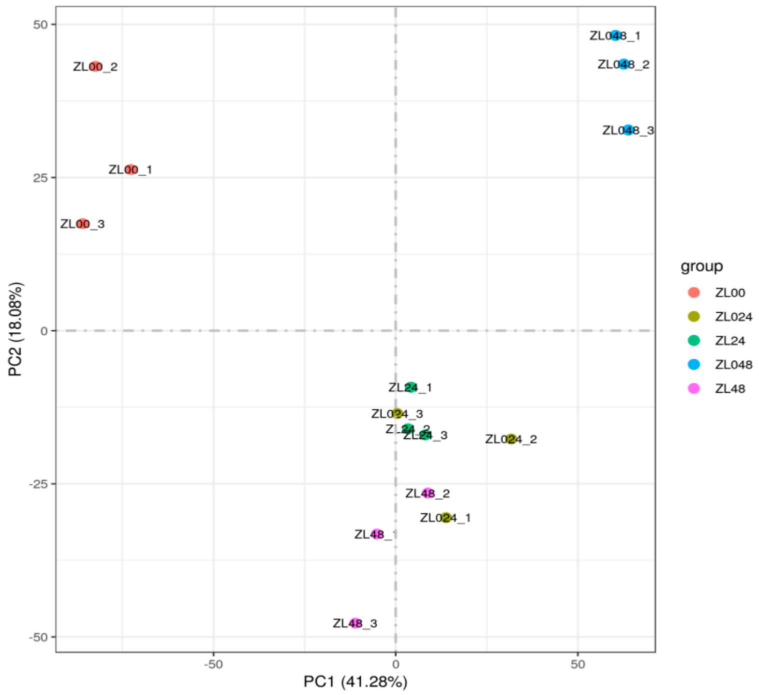


**Figure S1.** Principal component analysis of different samples. The horizontal coordinate represents the first principal component, whereas the longitudinal coordinate represents the second principal component. ZL00-1, ZL00-2, ZL00-3: the group at 0 h; ZL024-1, ZL024-2, ZL024-3: the control group at 24 h (three repetitions); ZL048-1, ZL048-2, ZL048-3: the control group at 48 h (three repetitions); ZL24-1, ZL24-2, ZL24-3: the treatment group (50 mg/L dihydrocoumarin) at 24 h (three repetitions); ZL48-1, ZL48-2, ZL48-3: the treatment group (50 mg/L dihydrocoumarin) at 48 h (three repetitions).

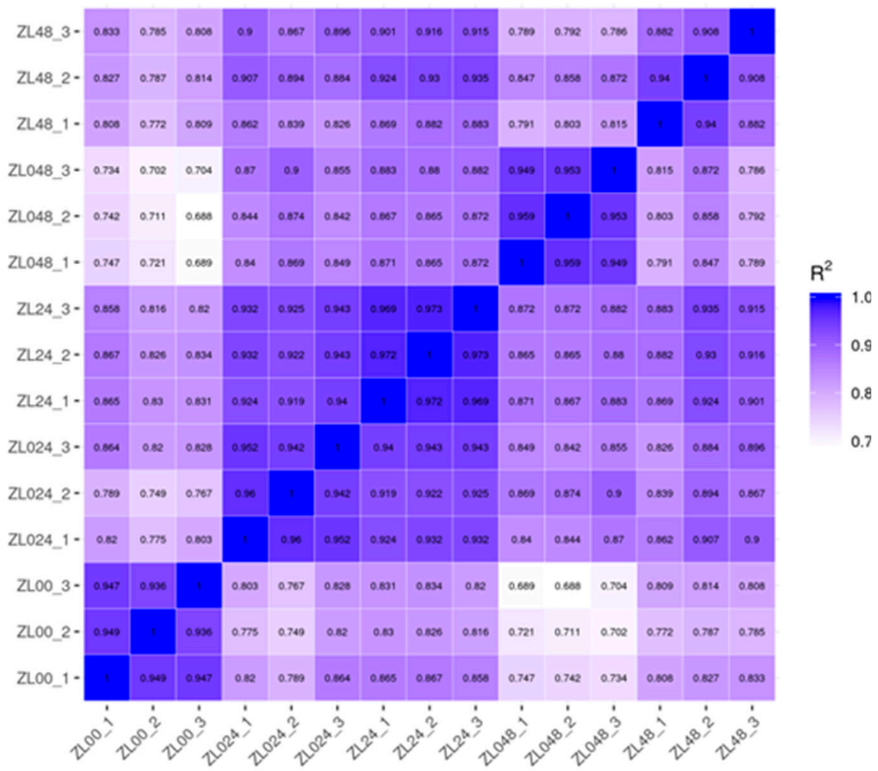


**Figure S2.** Pearson correlation heat map between different samples. The horizontal coordinate represents the squared correlation coefficients. ZL00-1, ZL00-2, ZL00-3: the group at 0 h; ZL024-1, ZL024-2, ZL024-3: the control group at 24 h (three repetitions); ZL048-1, ZL048-2, ZL048-3: the control group at 48 h (three repetitions); ZL24-1, ZL24-2, ZL24-3: the treatment group (50 mg/L dihydrocoumarin) at 24 h (three repetitions); ZL48-1, ZL48-2, ZL48-3: the treatment group (50 mg/L dihydrocoumarin) at 48 h (three repetitions).

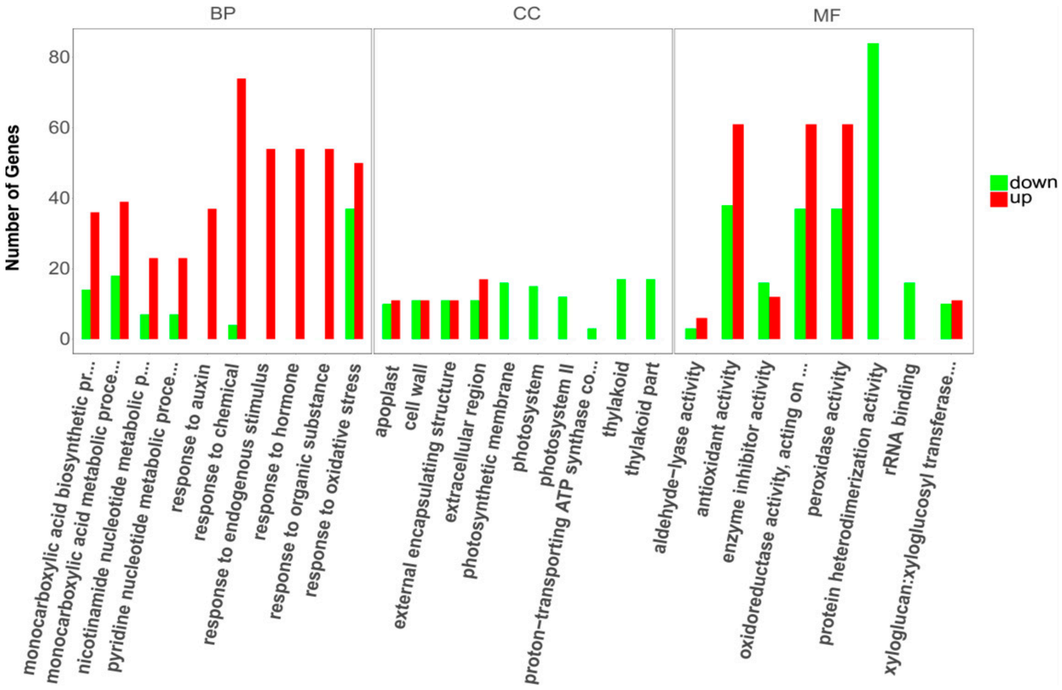


**Figure S3.** GO statistical histogram of differentially expressed genes between ZL24 and ZL024 under dihydrocoumarin treatment.

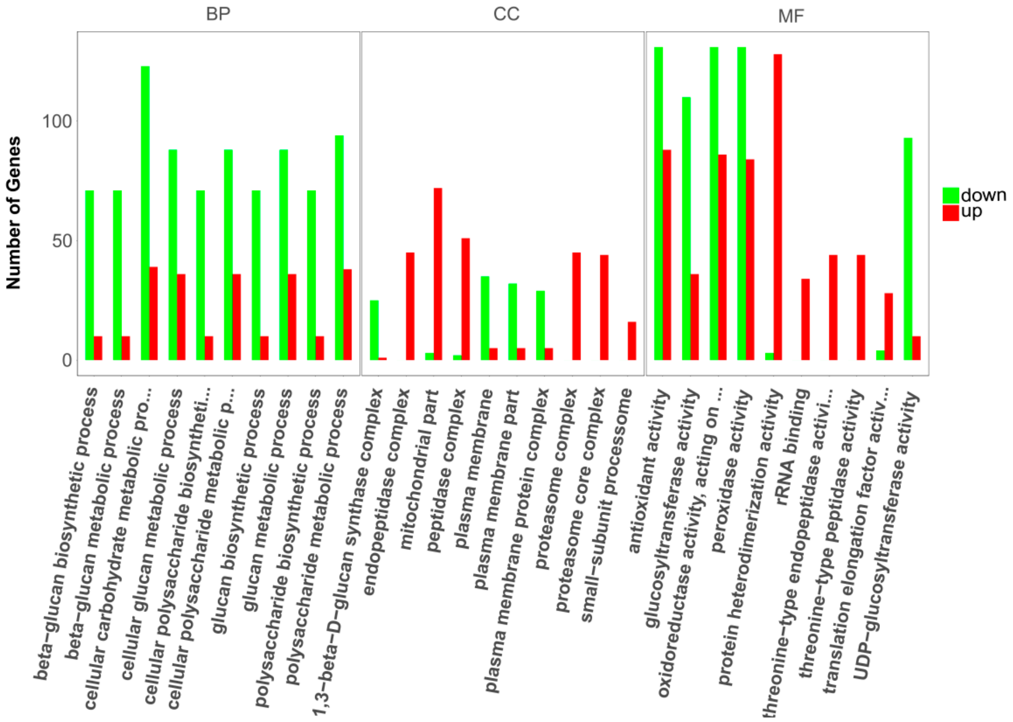


**Figure S4.** GO statistical histogram of differentially expressed genes between ZL48 and ZL048 under dihydrocoumarin treatment.

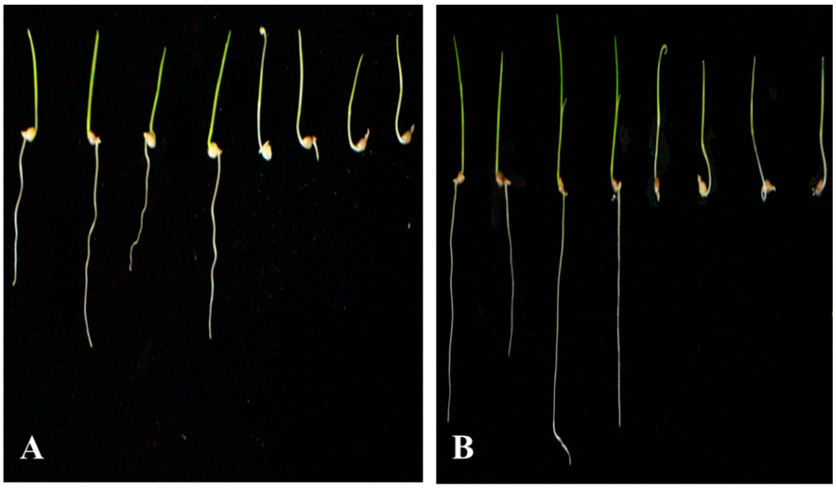


Figure S5: The barnyardgrass was treated with 50 mg/L dihydrocoumarin after 24 h (A) and 48 h (B).

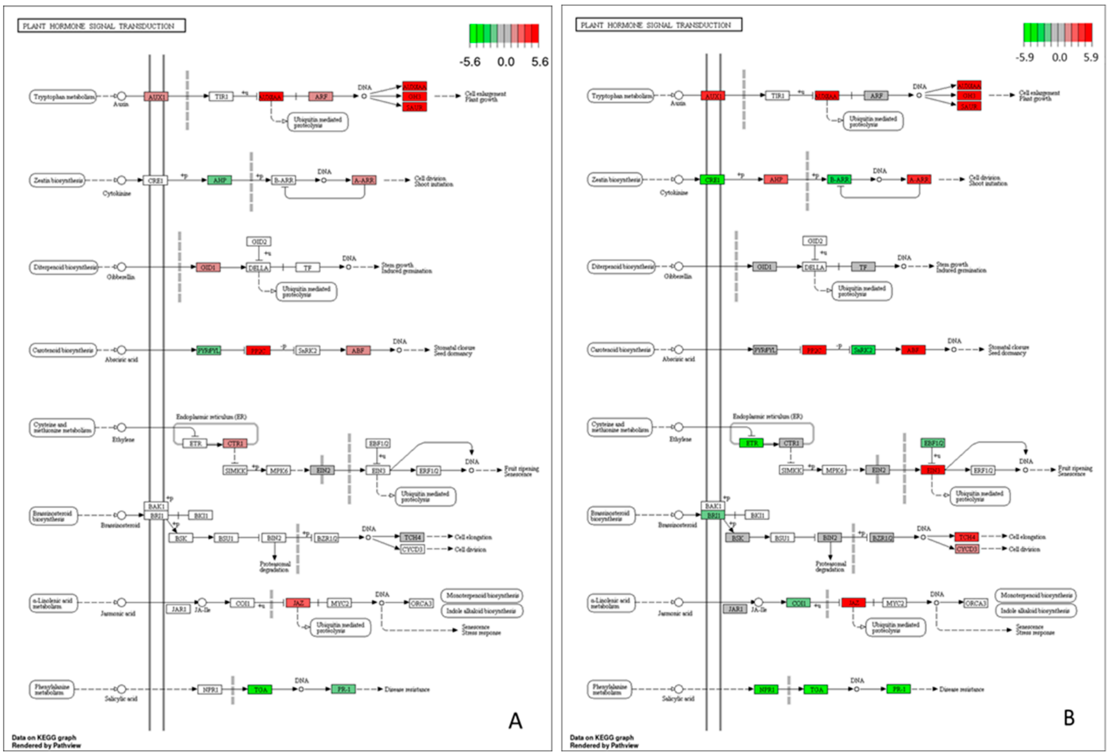


**Figure S6.** Plant hormone signal transduction KEGG pathways of barnyardgrass treated with dihydrocoumarin after 24 h (A)and 48 h (B).

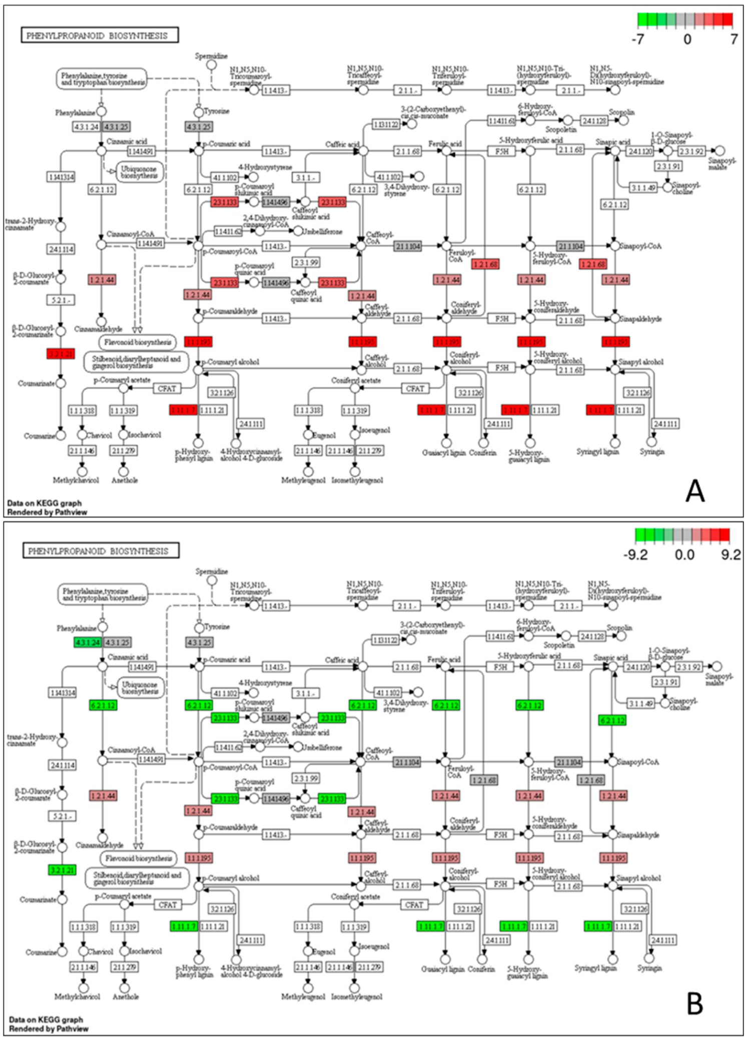


**Figure S7.** Phenylpropanoid biosynthesis KEGG pathways of barnyardgrass treated with dihydrocoumarin after 24 h (A)and 48 h (B).

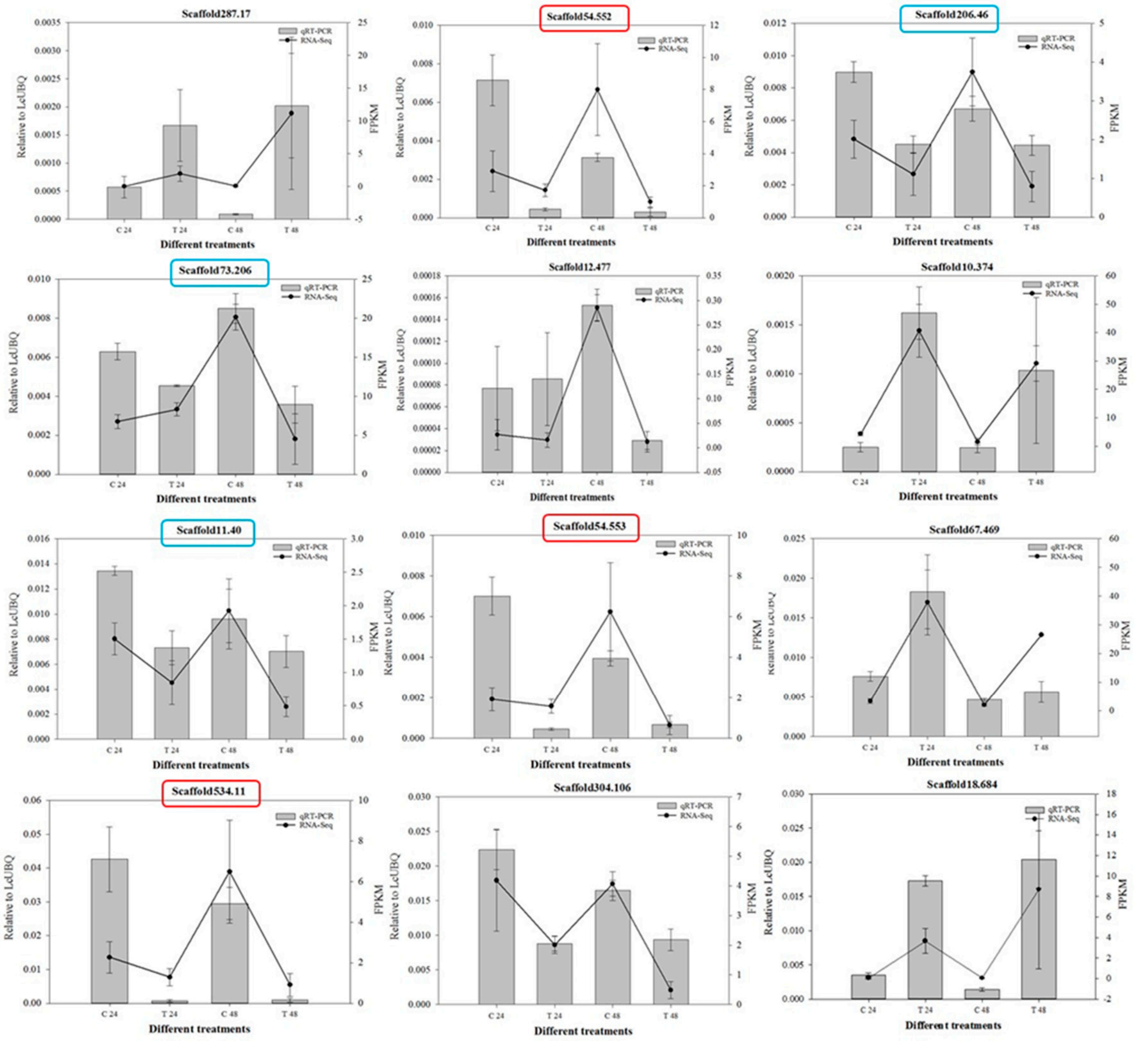


**Figure S8.** Expression of representative genes validated by qRT-PCR in barnyardgrass. T24 and T48 represent barnyardgrass treated with dihydrocoumarin for 24 h and 48 h; C24 and C48 are the controls; the PAL genes are surrounded by a red outline, whereas the 4CL genes are surrounded by a blue outline.
