# Supplementary material for: Interference of Dihydrocoumarin with Hormone Transduction and Phenylpropanoid Biosynthesis Inhibits Barnyardgrass (Echinochloa crus-galli) Root Growth
Source: Plants (Basel). 2022 Sep 26;11(19):2505. doi: 10.3390/plants11192505 (PMC9572682; doi:10.3390/plants11192505)
Supplement: Supplementary file 1 [file plants-11-02505-s001.zip › Respond Comments from Academic Editor and R1(the second round report).pdf]

Comments from Academic Editor (please provide point to point file)

Point 1: In their manuscript, the authors aim at characterization of dihydrocoumarin on barnyard grass root development. In fact treatment with the compound resulted in dosage-dependent inhibitory effects on root growth, substantially more pronounced than in rice. These observations do have some potential agronomic implications, as one might consider dihydrocoumarin as a potent bioherbicide for rice cultivation.

Response 1: Thank you for your affirmation, and our findings suggest that dihydrocoumarin can be used to control barnyardgrass growth in rice transplanting fields. We believe that the findings of this study are relevant to the scope of *Plants* and will be of interest to its readership.

Point 2: Approaches aimed at a characterization of molecular mechanisms by dihydrocoumarin might affect barnyard grass involved assessment of ROS production and transcriptome analysis. Based on their results the authors concluded that dihydrocoumarin would impact on hormonal signaling as well as on phenylpropanoid biosynthesis, which in turn might give rise to root growth inhibition. It is not entirely clear to me, how the authors came to this conclusion. In fact, when going through date presented in Figure 2 (i.e. cell death/membrane permeability/ROS production), it seems that conditions used for transcriptomics do have some very drastic consequences on cell viability in general. In other words: It is very well possible that adjustments in hormonal signaling/phenylpropanoid biosynthesis might contribute to dihydrocoumarin effects on root growth. However, it seems equally plausible that dihydrocoumarin treatment primarily affects cellular integrity (as indicated by ROS production and the drastic changes in membrane permeability caused by the compound), with the observed alterations in gene expression profiles, only arising as a consequence of rather general stress effects. This needs to be addressed, since otherwise any conclusion that is based on the outcome of the transcriptome analysis is merely anecdotal.

Response 2: Thank you for your suggestion, and it's very useful for us to improve our manuscript. To explain the validity of the treatment in transcriptomic, we have supplied a supplemental figure (Figure S5) to show the seedlings for transcriptomics analysis. The growth of root was seriously inhibited under the treatment of 50 mg/L dihydrocoumarin, and the shoot was not significantly inhibited compared with the controls, which was feasible to explore the mechanism of dihydrocoumarin affecting the growth of barnyardgrass root.

The treatment of Figure 2 was different from that in transcriptomic, and we chose the barnyardgrass seedlings with 1 cm root as the recipient to study the effects of dihydrocoumarin on barnyardgrass root, and the seedlings have grown in water 3-4 d. The results showed that dihydrocoumarin treatment could cause oxidative stress in barnyardgrass, disrupt the cell membrane and reduce the root cell activity, resulting in root cell death. The phenotype of barnyargrass root echoed the physiological results which indicated that the action mechanism of dihydrocoumarin was associated with root development. Hence, according to the comprehensive analysis of KEGG and GO enrichment results, combined with the physiological results and DEGs, we further analyzed the plant hormone signal transduction pathway and phenylpropanoid biosynthesis pathway of barnyardgrass seedlings after dihydrocoumarin treatment.

Point 3: Enzyme activity assays on display in Figure 5C are not accurately described. Do the repetitions correspond to biological or technical repeats? Which protocol has been used for the assays? Which statistical tools have been used for data analysis? It seems quite remarkable that, regardless of dihydrocoumarin treatment, enzymatic activities in all the assays do show a quite uniform trend - an increase after 72 hours followed by a decrease after 120 hours of treatment. Is there an explanation for this observation?

Response 3: We are sorry for the confusion, and we have described more details on the enzyme activity assays of Figure 5C. The activity of the enzymes PAL and 4CL first increased and then decreased under both control and dihydrocoumarin treatments, indicating that the addition of 50 mL water and dihydrocoumarin probably stimulated the activities of PAL and 4CL to resist stress. The increase in enzyme activities was higher after dihydrocoumarin treatment than after the control treatment, which suggests that the enzyme activities were probably suppressed by dihydrocoumarin at the same detection time points.

The repetitions and data analysis method has been described in "4.10 Statistical analysis": Three biological replicates were collected from every treatment group, and the data are presented as the mean ± standard error for three replicates. Statistical analysis was conducted using SPSS (v. 26.0) software. One-way analysis of variance was used for comparison between the groups, and the significance was determined using the Duncan's multiple range test. A P value of <0.05 was considered to denote the statistical significance.

Point 4: The discussion section, describing the outcome of the transcriptomics analyses is somewhat confusing and requires some accurate editing/polishing. E.g. lines 317-321; 330-332; 334-336; etc.

Response4: We are very sorry for our incorrect writing, and the language has been carefully polished by a professional service (TopEdit) according your suggestions, and also the discussion section of the transcriptomics analyses. These revisions are highlighted in red, and special thanks to you for your good comments.

Comments from R1 – second round report (please provide point to point file)

Point 1: Authors have addressed the majority of the issues raised. However, there is still space for further improvement of the manuscript, especially towards revealing novelty and originality of the study and highlighting its practical use.

Response 1: Thank you. Dihydrocoumarin was the main allelochemicals of the decomposition of *Myosoton aquaticum* (L.) Moench was identified by us firstly, and there was no other research about the allelopathy of dihydrocoumarin before. We had proved that dihydrocoumarin could be used as a potential pre-emergence herbicide in rice transplanting fields, and speculated that dihydrocoumarin regulated the hormone signal transduction and phenylpropanoid biosynthesis pathways, which eventually affected barnyardgrass growth. We hope the study could provide new

thinking for the development of allelochemicals, and provide a theoretical basis for the research of botanical herbicides.

Special thanks to you for your good comments!

Thank you very much for your time and suggestions!

Once again, thanks for the academic editor and reviewers' comments and suggestions.

Wish you all the best!

Yours sincerely,

Haona Yang
